# Does Spontaneous Favorability to Power (vs. Universalism) Values Predict Spontaneous Prejudice and Discrimination?

**DOI:** 10.1111/jopy.12269

**Published:** 2016-08-10

**Authors:** Nicolas Souchon, Gregory R. Maio, Paul H. P. Hanel, Brigitte Bardin

**Affiliations:** ^1^ Université de Paris Ouest; ^2^ Cardiff University; ^3^ University of Toulouse

**Keywords:** human values, prejudice, implicit measures, power

## Abstract

**Objective:**

We conducted five studies testing whether an implicit measure of favorability toward power over universalism values predicts spontaneous prejudice and discrimination.

**Method:**

Studies 1 (*N* = 192) and 2 (*N* = 86) examined correlations between spontaneous favorability toward power (vs. universalism) values, achievement (vs. benevolence) values, and a spontaneous measure of prejudice toward ethnic minorities. Study 3 (*N* = 159) tested whether conditioning participants to associate power values with positive adjectives and universalism values with negative adjectives (or inversely) affects spontaneous prejudice. Study 4 (*N* = 95) tested whether decision bias toward female handball players could be predicted by spontaneous attitude toward power (vs. universalism) values. Study 5 (*N* = 123) examined correlations between spontaneous attitude toward power (vs. universalism) values, spontaneous importance toward power (vs. universalism) values, and spontaneous prejudice toward Black African people.

**Results:**

Spontaneous positivity toward power (vs. universalism) values was associated with spontaneous negativity toward minorities and predicted gender bias in a decision task, whereas the explicit measures did not.

**Conclusions:**

These results indicate that the implicit assessment of evaluative responses attached to human values helps to model value‐attitude‐behavior relations.

Famous people throughout history have shown intense attachment to power while denying that they like power. For example, although Napoleon ascended from a low status in Corsica to emperor of France, he felt more attached to high ideals of honor, liberty, and equality than to power (Cronin, 1971). Centuries before Napoleon, Niccolò Machiavelli's (1513/1981) classic treatise, *The Prince*, suggested that successful rulers must place power above virtue, occasionally using brutality and vice as tools, while not revealing their singular obsession with power to others. These examples show how either a sincere belief in self‐transcending values (e.g., equality) or a mere desire to *seem* virtuous may lead power‐accruing individuals to *claim* that power is unimportant to them. Yet, to others, they may look more like paragons of power than paragons of virtue. Importantly, such discrepancies may easily arise for other abstract values, such as freedom, equality, or helpfulness. People can vary in how much importance they consciously ascribe to these ideals, and it is an open question whether their conscious ascriptions reflect how they feel about the values at a deeper level. The present research examines this issue. Focusing on power and equality in particular, we test whether implicit measures can contribute to the assessment of values.

## Values and Attitudes

Classic and contemporary measures of values have relied on self‐report measures, which ask participants to reflect on the relative importance of different values (Rokeach, 1973; Schwartz, 1992). For instance, Rokeach's (1973) classic measure asks participants to thoughtfully rank‐order the importance of 36 values (in two sets). In the most popular contemporary measure, the Schwartz Value Survey (Schwartz, 1992), participants are instructed to scan a list of values, note which ones are least and most important, and then rate the importance of each value as a guiding principle in their life. Also, in a more recent measure, the Portrait Value Questionnaire (PVQ; Schwartz et al., 2001), participants read short portraits describing a person's goals and rate their own similarity to the person.

Self‐reports are also the dominant method for assessing attitudes. Attitudes are commonly defined as tendencies to evaluate an attitude object with some degree of favor or disfavor, and an attitude object can be any concrete or abstract entity that we like or dislike (Haddock & Maio, 2015). Long‐standing theory and research has supported the idea that there are strong, basic connections between values and attitudes. Theories of attitude function (i.e., the psychological needs that attitudes fulfill; e.g., Herek, 1986; Katz, 1960) indicate that we can shape our attitudes to reflect our personal values, and this has important ramifications for predicting attitude change (Blankenship & Wegener, 2008) and behavior (e.g., Maio & Olson, 1995). This view is also commensurate with many studies revealing links between values and attitudes (see Maio & Olson, 2000, for a review).

Nevertheless, research on attitudes in the past two decades has been greatly enhanced by the inclusion of implicit measures. As described by Fazio and Olson (2003), implicit measures of attitude tap spontaneous evaluations that are activated without control and deliberation. Fazio and Olson suggest that implicit measures assess the same attitude as explicit, self‐report measures, but the implicit measures yield a snapshot of the spontaneous evaluations that may feed into later, downstream conscious reports of attitudes. It is *not* the case that the constructs assessed by implicit measures are any more “real” than the attitudes assessed by explicit measures or that they have a “truth value” (e.g., Greenwald, Poehlman, Uhlmann, & Banaji, 2009; Hofmann, Gawronski, Gschwendner, Le, & Schmitt, 2005; Nosek et al., 2007; Strack & Deutsch, 2004); implicit measures simply assess the components of attitude that are not subject to deliberation during the measurement procedure. Thus, implicit and explicit measures of attitude are often correlated because they assess the same attitude, but the magnitude of the correlation depends on the extent to which conscious processing moves people away from the spontaneous evaluations (e.g., Gawronski & Bodenhausen, 2006). Thus, both measures together are more useful than either measure alone.

An interesting issue is whether the contemporary, explicit self‐report measures of values are sufficient for predicting scores on *both* types of measures of attitude. While there is abundant evidence about the utility of contemporary measures of values for predicting responses on explicit measures of attitude (Maio & Olson, 2000), evidence about their ability to predict implicit measures of attitude is lacking. This is an interesting omission because the current methods for assessing values rely on careful thought in responding, whereas the implicit measures of attitude capture spontaneous evaluations of an attitude object. It has been suggested that these spontaneous evaluations are more strongly associated with other spontaneous judgments and actions than with more deliberate judgments and action (e.g., Dovidio, Kawakami, Johnson, Johnson, & Howard, 1997; Fazio & Olson, 2003). This reasoning suggests that spontaneous evaluations of attitude objects should be more strongly associated with spontaneous judgments of values–both of which escape the complex cognitive filtering that occurs during deliberative responses to explicit measures.

Spontaneous judgments of values may be important also because of the psychological components of values. Values tend to be more affectively based than cognitively based (Maio & Olson, 1998; see Maio, 2010, for a review), and mere value priming, even without deliberative judgments of value importance, affects relevant value judgments and behavior (Maio, Pakizeh, Cheung, & Rees, 2009; Pakizeh, Gebauer, & Maio, 2007). Given the existence of psychological obstacles to inferring our goals (e.g., Ferguson & Porter, 2010) and feelings (e.g., Schwarz & Clore, 1997), it may be difficult for people to identify these contributions in explicit measures of values. Indeed, such difficulties also occur even when inferring simpler attitudes and our bases for them (Wilson, Lindsey, & Schooler, 2000). By circumventing this introspective difficulty and social desirability, implicit measures have the potential to uncover facets of values that are undetected in explicit measures.

How might we assess these spontaneous judgments of values? A long‐standing tradition has measured motivational disposition by coding imaginative thought in stories written about people in social situations, like a “captain speaking to a passenger” or “persons seated on a park bench” (McClelland, Koestner, & Weinberger, 1989; Schultheiss, 2008; Winter, John, Stewart, Klohnen, & Duncan, 1998). Similar to this approach, recent research has coded values from text in interviews (e.g., Frimer, Walker, Dunlop, Lee, & Riches, 2012). However, this approach requires training, large amounts of time for measurement (e.g., 2‐hour interviews), and considerable labor in coding. More important, this approach differs in many ways from contemporary self‐report measures (e.g., numerical scaling, measurement structure, role of deliberation), making it difficult to know precisely how they differ from self‐reports. In contrast, implicit measures of attitude can be more closely linked in content to explicit measures of attitude. Furthermore, given the aforementioned evidence linking implicit measures of attitude *and* self‐report measures of values to affective processes, these implicit measures may tap important psychological components of values.

The Implicit Association Test (IAT; Greenwald, McGhee, & Schwartz, 1998) is the most commonly used paradigm for implicit measurement. Notwithstanding some important limitations and controversies (e.g., Fiedler, Messner, & Bluemke, 2006; Teige‐Mocigemba, Klauer, & Sherman, 2010), numerous studies have demonstrated the predictive validity of the IAT (see Greenwald et al., 2009, for a meta‐analysis; Perugini, Richetin, & Zogmaister, 2010). In the applications relevant to our interest in implicitly measuring values, researchers have adapted this test by presenting stimuli that were theoretically related to implicit needs or motives, finding evidence for predictive validity using both a verbal (Sheldon, King, Houser‐Marko, Osbaldiston, & Gunz, 2007) and a Pictorial Attitude IAT (PA‐IAT; Slabbinck, De Houwer, & Van Kenhove, 2011). For example, the PA‐IAT for power presents images related to “power” or “nonpower” as target stimuli and words related to “attractive”(e.g., *nice*, *friendly*) versus “non‐attractive”(e.g., *creepy*, *unpleasant*) as evaluative categories. Unlike our proposed implicit measurement of values, however, these tests are focused on abstract motives and are not grounded in values theory. For example, Sheldon et al. (2007) contrast power with intimacy, which is similar to the opposition between power and universalism values in Schwartz's (1992) model, but conceptually distinct. Also, Slabbinck and colleagues (2011) focus on attractiveness associations with social connotations (e.g., *friendly*, *creepy*) that are distinct from the personal connotations we aim to tap. Nonetheless, these approaches provide evidence that it may be useful to adapt an IAT to the assessment of values within Schwartz's model (1992), as we sought to do in the present research.

## Power and Prejudice

Implicit measures are particularly useful in socially sensitive domains, as in research using implicit measures to examine prejudice (e.g., Dovidio, Kawakami, & Gaertner, 2002). Prejudice is particularly relevant to individual differences in power versus universalism values. In over 80 nations, Schwartz (1992) has found that power and universalism values are on opposing ends of the same motivational continuum; the rated importance of power values is negatively correlated with the rated importance of universalism values. Abundant social psychological theory and research has documented how people who prefer status or power over equality and social justice are more likely to exhibit prejudice (e.g., Duckitt, Wagner, du Plessis, & Birum, 2002; Sidanius & Pratto, 1999). The present research therefore examines the utility of implicit measures of attitudes toward power versus universalism values in this context.

For this purpose, we developed the Attitudes toward Values IAT (AV‐IAT) to assess spontaneous attitudes toward power versus universalism values. We did not conceptualize these spontaneous attitudes as measuring the *real* values, but rather as tapping spontaneous components of values. By taking this approach, the AV‐IAT is able to look directly at the role of evaluative associations with values, but it does not look at spontaneous relative differences in value *importance*. We were uncertain whether an IAT can reliably assess spontaneous differences in importance (which involves weighting an object relative to a goal), and our final study tackles this question.

As important test cases, we focused on attitudes toward people of different ethnicity and gender. After examining the AV‐IAT in connection to these spontaneous attitudes, we examined its ability to predict spontaneous discrimination, focusing on bias against women in particular. We expected that higher levels of spontaneous favorability to power over universalism (as recorded in the AV‐IAT) would significantly predict (a) more negative attitudes toward people of outgroup ethnicity and out‐group gender (as recorded using paper‐and‐pencil or computer IATs) and (b) more discrimination against women. Moreover, we expected that these relations would be unique, occurring over and above the associations with self‐report measures of attitudes toward values. That is, instead of asking participants to rate the importance of the values (as in standard values questionnaires), we asked participants to rate their favorability to the values included in the implicit measure of attitude to values. This enabled greater comparability between the two levels of measurement and a sterner test of the AV‐IAT's discriminant validity. If we were to contrast the AV‐IAT with the contemporary measures of values, these measures would have differed in terms of both the type of judgment (i.e., evaluation vs. importance) and the level of measurement (i.e., implicit vs. explicit). In contrast, the AV‐IAT and explicit measures of attitude toward values focus on the same type of judgment, making it implausible that any differences in predictive utility between these measures could be attributable to the judgment dimension alone. Nonetheless, our final study followed up this test with a less conservative test of whether the AV‐IAT and a value importance IAT both exhibit predictive validity beyond contemporary measures of values.

## Study 1

Study 1 tested whether the implicit measurement of attitudes toward values can help to predict attitudes toward ethnic groups. In one sample, we used the power versus universalism values AV‐IAT to predict attitudes toward Arab people, a prominent ethnic out‐group in France, where Study 1 was conducted. Studying stereotypes and prejudice toward Arab people is particularly relevant for historical reasons in France. In this nation, spontaneous stereotypes (Chateignier, Dutrévis, Nugier, & Chekroun, 2009) and attitudes toward Arab people tend to be strongly negative (Dambrun & Guimond, 2004). In a separate sample, we used the power versus universalism values AV‐IAT to predict attitudes toward Black African people. Prejudice toward this ethnic group is a powerful test case because abundant research has shown prejudice against this group using both explicit and implicit measures (e.g., Dovidio et al., 2002; Wittenbrink, Judd, & Park, 2001).

Across both target groups, Study 1 tested whether the ethnic in‐group/out‐group bias shown in IATs was predictable from differences in spontaneous evaluations of power and universalism values. Moreover, because of positive relations between power values and achievement values and between universalism values and benevolence values (see Schwartz, 1992), we also wished to test whether any effects of an implicit measure of attitudes toward power versus universalism values occurred independently of an implicit measure of attitudes toward achievement versus benevolence values. Distinct effects of the implicit measure of attitudes toward power‐universalism values would support the hypothesis that these values are uniquely connected to the in‐group versus out‐group bias, and such evidence would provide further testimony to the validity of the new implicit measures of attitudes toward values. Study 1 also provided a further test of the discriminant validity of the AV‐IAT, by using it to predict implicit ethnic in‐group bias independently of explicitly reported attitudes to values.

#### Method

##### Participants

###### Sample for Prediction of Prejudice Against Arabs

Ninety‐five participants (21 women; *M*_age_ = 21.58, *SD* = 1.98) completed the study in groups of 15 to 20 during lectures. All participants were of French nationality, but 15 participants had at least one parent who was born in Morocco, Tunisia, or Algeria, and seven additional participants had at least one grandparent from one of these countries.

###### Sample for Prediction of Prejudice Against Black Africans

Ninety‐seven participants (27 women; *M*_age_ = 28.23, *SD* = 14.22) completed the study in groups of 3 to 20 within university. All participants were of French nationality, but four participants had at least one parent originating from a Black African country,^1^ and one additional participant had at least one grandparent from one of these countries.

##### Procedure

Participants were told that they were taking part in a study of personality. They completed a flower‐insect paper‐and‐pencil IAT as practice before completing a power‐universalism AV‐IAT, an achievement‐benevolence AV‐IAT, and a paper‐and‐pencil IAT for the target group of interest (i.e., Arabs or Black Africans). The appendix lists the items within the IATs (Table 1). Participants then completed explicit measures of attitudes toward power, universalism, achievement, benevolence, French people, and either Arabs or Black Africans. Finally, participants were debriefed.

**Table 1:** Descriptive Statistics and Correlations for the Implicit Measures Across the FiveStudies

		ND	*D*	*SD*	*t*(0)	95% CI	2	3
Study 1	Pow‐U AV‐IAT	17.57%	–.94	2.95	−3.74*	[–1.45, –.44]	.60**	.27**
	Achi‐B AV‐IAT	16.36%	–.52	2.95	−2.07*	[–1.02, –.02]		.17*
	Race IAT	4.24%	2.15	2.24	12.05**	[1.79, 2.50]		–
	Arab IAT	2.73%	2.01	2.45	6.9**	[1.82, 2.70]		
	Black IAT	5.04%	2.26	2.06	10.2**	[1.42, 2.58]		
	**Sum**	12.7%						
Study 2	Pow‐U AV‐IAT	6.9%	–.30	.52	−5.13**	[–.42, –.18]	.43**	.28*
	Achi‐B AV‐IAT	4.65%	–.15	.47	−2.94**	[–.25, –.05]		.02
	Black IAT	3.4%	.38	.37	9.17**	[.29, .46]		–
	**Sum**	5.03%						
Study 3	Black IAT Pow +	1.8%	2.48	2.47	7.23**	[1.79, 3.17]		
	Black IAT Control	5.6%	1.71	2.07	5.83**	[1.12, 2.30]		
	Black IAT Uni +	7.6%	1.11	2.10	3.65**	[.49, 1.71]		
	**Sum**	5.6%	1.78	2.28	9.57**	[1.41, 2.15]		
Study 4	Pow‐U AV‐IAT	37.8%	–.68	2.78	−1.87	[–1.41, .04]	.24	.31*
	Achi‐B AV‐IAT	31.5%	–.34	2.62	−1.04	[–1.0, .31]		–.02
	Gender IAT	7.3%	1.81	1.67	10.14**	[1.45, 2.16]		–
	**Sum**	25.6%						
Study 5	Pow–U AV–IAT	22.76%	−1.66	2.84	−5.71**	[–2.24,–1.08]	.46**	.24*
	Pow–U ImpIAT	17.88%	–.44	2.35	−1.89+	[–.91, .02]		.27**
	Black IAT	5.69%	1.85	2.30	8.66**	[1.43, 2.27]		
	**Sum**	15.44%						

*Note*. ND = number of deletions; *D* = *D*–score calculated according to Lane et al. (2005) for Studies 1, 3, and 4 and Greenwald et al. (2003) for Study 2; *t*(0) = *t*–test from 0; 95% CI = 95% confidence interval; Pow–U = power–universalism; Achi–B = achievement‐benevolence; Imp = importance.*p < .05. **p < .01.

Paper‐and‐pencil IATs are based on the original computerized version of the IAT. The paper‐and‐pencil version calculates scores using timed classifications on paper instead of reaction times on computer. Both versions are in wide use, and they reveal similar effects (e.g., Sekaquaptewa, Vargas, & Von Hippel, 2010; Vargas, Sekaquaptewa, & Von Hippel, 2007). However, the paper‐and‐pencil IAT is easily presented to large groups and is less costly to implement.

##### Measures

###### Flower‐Insect IAT

For practice, we first gave participants a paper‐and‐pencil IAT assessing favorability toward flowers versus insects. The measure presented columns of 24 words on different pages. Participants chose whether words such as *daisies*, *tulips*, *bugs*, and *mosquitoes* belonged to the concept category “flowers” or “insects,” and whether words such as *wonderful*, *joyful*, *terrible*, or *nasty* belonged to the evaluative category “good” or “bad.” The concept and evaluative categories were simultaneously paired in two configurations. In the first configuration (first page), the categories “flowers‐good” (top left side) and “insects‐bad” (top right side) were paired, whereas in the second configuration (second page), the categories “flowers‐bad” (top left side) and “insects‐good” (top right side) were paired. We counterbalanced the order of the configurations across participants.

Participants worked their way down the column of words, placing a checkmark in a response bubble to the left or right of each word to indicate the column category to which the word belonged. Participants were asked to work as quickly and accurately as possible, but to continue without stopping if mistakes occurred. Participants were given 20 seconds to classify as many words as possible on each page. Participants were then asked to note the new category pairings on the second page before the timed classification task began again.

###### Power Versus Universalism and Achievement Versus Benevolence AV‐IAT

The AV‐IAT for assessing attitudes toward power over universalism values and achievement versus benevolence values used the same structure as the practice IAT, except that the stimulus words were related to power values (e.g., *authority*, *wealth*), universalism values (e.g., *equality*, *social justice*), achievement values (e.g., *ambitious*, *successful*), or benevolence values (e.g., *forgiving*, *helpful)*. The value words were adapted from the Schwartz Value Survey (Schwartz, 1992), and the adjectives were the same as those in the practice paper‐and‐pencil IAT.

###### French People Versus Arab or Black African People IAT

The paper‐and‐pencil IAT assessing attitudes to French versus Arab people or French versus Black African people used the same structure as the flowers versus insects IAT, except that the stimuli included words (i.e., first name) that were related to “French people,” “Arab people,” and “Black African people.”

###### Scoring

For each implicit measure, the variable of interest was the difference in the number of correctly classified items under the two category pairings. Participants generally classify the stimuli faster when the paired categories match their automatic attitudes toward the category (e.g., “flowers” paired with *good* and “insects” paired with *bad*) than when they are mismatched. Before calculating the IAT scores, we followed prior research by first excluding participants who failed to classify at least eight items per page and who made more than 20% errors by page (e.g., Lane, Mitchell, & Banaji, 2005). Scores were then calculated as ±[maximum/minimum]*√(maximum‐minimum), where *maximum* is the number of correctly categorized items on the block for which participants completed more correct items, and *minimum* is the number of items correctly categorized on the block for which they completed fewer correct items. This algorithm is based on analyses of simulated data sets that mirror the distribution of general IAT effects. According to Lane et al. (2005), this algorithm (a) best accounts for the difference between the number of items completed and individual differences in speed in completing categorization tasks in general, (b) minimizes the influence of extreme scores, and (c) reduces the overall skewness of the data distribution.

In the analysis of the French people–out‐group ethnicity paper‐and‐pencil IAT, we multiplied scores by −1 if the maximum scores arose from the French people–negative and out‐group ethnicity–positive block, thereby making higher scores indicate more positivity to French over out‐group ethnicity people. In the analysis of the power‐universalism AV‐IAT and achievement versus benevolence AV‐IAT, we multiplied scores by −1 if the maximum scores arose from the power‐negative and universalism‐positive block, or achievement‐negative and benevolence‐positive block, thereby making higher scores indicate more positivity to power values and more negativity to universalism values or more positivity to achievement values and more negativity to benevolence values.

###### Explicit Measures

Participants were asked to rate their feelings about power, universalism, achievement, benevolence, Arab people, Black African people, or French people using 7‐point semantic differential scales ranging from −3 (*bad*) to +3 (*good*). Each values category was described using three values items representing the category (e.g., social justice, equality, and broad‐mindedness for universalism values).

#### Results and Discussion

After excluding Arab and Black African descendants (because of their relevance to our measures of prejudice), we combined the subsamples (*N* = 165) by creating measures of French‐national versus ethnic out‐group attitude. We did this for three reasons: (a) the variable we were interested in was “out‐group attitude,” and each group was a similar instance of an ethnic out‐group; (b) this combination improves power; and (c) the power‐universalism AV‐IAT predicts implicit prejudice in each group separately. Furthermore, a regression analysis including a dummy code to represent the ethnicity of the target alongside the implicit predictors, model *R* ^2^ = .13, *F*(7, 109) = 2.29, *p* = .03, revealed no significant interactions with the dummy code (βs = –.06 to .15, *p*s > .59). Table 1 shows the exclusions that resulted from applying the recommended exclusion criteria for the paper‐and‐pencil IATs.^2^

##### Implicit Measures

Table 1 presents descriptive data for the IATs. The *D*‐scores show that participants in this study exhibited significantly less spontaneous favorability to power than to universalism and less spontaneous favorability to achievement than to benevolence. Furthermore, congruent with Schwartz's (1992) model, participants who were spontaneously more favorable to power (vs. universalism) values were also more likely to show greater spontaneous favorability to achievement (vs. benevolence) values. Also, participants were more negative to the ethnic out‐groups than to French people.

##### Explicit Measures

Table 2 presents the descriptive statistics for the explicit measures. Participants explicitly evaluated power less favorably than universalism, *t*(164) = −10.06, *p* < .001, and judged achievement as favorably as benevolence, *t*(164) = −1.14, *ns*. Participants were more favorable to people of French origin than to the ethnic out‐groups, *t*(164) = 2.26, *p* < .03. In addition, congruent with Schwartz's (1992) model, participants who evaluated power more favorably also evaluated achievement more favorably, and participants who evaluated universalism more favorably evaluated benevolence more favorably (see Table 3).³

**Table 2:** Study 1: Descriptive Statistics for Explicit measures

Attitude Measures	ND	α	*M*	*SD*	95% CI
Power values	0	.91	.48	1.15	[.30, .66]
Universalism values	0	.92	1.59	.89	[1.46, 1.73]
Power‐universalism	0	–	−1.11	1.42	[–1.33, –.89]
Achievement values	0	.94	1.95	.98	[1.80, 2.10]
Benevolence values	0	.93	2.05	.87	[1.92, 2.19]
Achievement‐benevolence values	0	–	–.10	1.10	[−.26, .07]
French	0	.96	.78	1.01	[.63, .94]
Arab	0	.95	.53	.98	[.30, .76]
Black African	0	.96	.69	1.01	[.48, .90]
Out‐group	0	.95	.62	1.01	[.47, .77]
Fr‐Out‐group	0	–	.16	.93	[.02,.31]

*Note*. ND = number of deletions or missing data; 95% CI = 95% confidence interval.

**Table 3:** Study 1: Correlations Between Implicit and Explicit Measures

	1	2	3	4	5	6	7	8	9	10	11	12
1. PU IAT	–	.60**	.27**	.33**	−.21*	.40**	.24**	−.09	.30**	.04	−.09	.13
2. AB IAT		–	.17*	.24*	.005	.19*	.36**	.01	.32**	.11	.14	−.02
3. Race IAT			–	.01	−.11	.08	.09	−.05	.13	.08	−.15+	.23**
4. Power				–	.05	.77**	.42**	−.11	.46**	−.09	−.11	.02
5. Uni					–	−.58**	.16*	.46**	−.21**	.12	.27**	−.16*
6. Pow‐Uni						–	.24*	−.37**	.51**	−.15+	−.27**	.12
7. Achi							–	.29**	.65**	.15+	.10	.05
8. Ben								–	−.53**	.23**	.25**	−.01
9. Achi‐Ben									–	−.05	−.10	.05
10. French										–	.56**	.47**
11. Out‐group											–	−.45**
12. Fr‐Out												–

*Note*. PU IAT = power‐universalism AV‐IAT; AB IAT = achievement‐benevolence AV‐IAT; Race IAT = attitude toward French versus ethnic out‐groups IAT; Power, Uni, Achi, and Ben = explicit measures of attitude toward power, universalism, achievement, and benevolence; French and Out‐group = explicit measures of attitude toward French people and the ethnic out‐group people. *N*s vary between 119 and 165 due to missing data on the power‐universalism AV‐IAT, the achievement‐benevolence AV‐IAT, and the attitude toward French versus ethnic out‐groups IAT.

+*p* < .06. *p < .05. **p < .01

##### Prediction of Prejudice

Table 3 presents the correlations among all our implicit and explicit measures. For the sake of brevity, we highlight the findings most pertinent to our predictions regarding power versus universalism. The results show that more spontaneous positivity to power (vs. universalism) on the AV‐IAT was associated with a spontaneous negative attitude to the ethnic out‐groups on the prejudice IAT. Looking at the explicit measures, participants who evaluated power more positively were not significantly more negative to the ethnic out‐groups than to French people, whereas ratings of universalism were associated with more positivity to the ethnic out‐groups.

A regression analysis, *R* ^2^ = .09, *F*(4, 112) = 2.77, *p* = .03, was conducted to test whether the power‐universalism AV‐IAT continued to predict spontaneous prejudice after controlling for achievement‐benevolence AV‐IAT scores and the explicit measures of attitudes toward power versus universalism and achievement versus benevolence. Results indicated that the implicit measure of attitudes toward power versus universalism continued to predict the French–out‐group IAT score (β = .24, *p* = .04, ${ŋ}_{p}^{2}$ = .04), but the implicit measure of attitudes to achievement versus benevolence (β = .06, *ns*), the explicit attitudes to power‐universalism score (β = –.12, *ns*), and the explicit attitudes to achievement‐benevolence scores did not (β = .11, *ns*). Thus, the power‐universalism AV‐IAT explained variance in spontaneous prejudice that was not explained by the explicit measures of attitudes to the values and the implicit and explicit measures of achievement versus benevolence values.

## Study 2

Study 1 provided provocative evidence for the utility of measuring values with an implicit measure, using the paper‐and‐pencil AV‐IAT to measure relative favorability to power versus universalism values. While paper‐and‐pencil IATs reveal effects that are similar to those found in computer IATs (e.g., Sekaquaptewa et al., 2010; Vargas et al., 2007), replication with a computer‐based IAT would provide convergent support. Thus, in Study 2, we assessed values and prejudice to Black African people using the original computer‐based IAT. No explicit measures were included because we primarily sought replication of the implicit associations.

#### Method

##### Participants

Eighty‐six participants (36 women; *M*_age_ = 30.10, *SD* = 11.38) completed the study individually. All participants were of French nationality without parents or grandparents originating from a Black African nation.

##### Procedure

Participants were told that they were taking part in a study of categorization under time pressure. They completed three IATs using Inquisit(web) software: a five‐word power‐universalism AV‐IAT, a five‐word achievement‐benevolence AV‐IAT, and a five‐word French versus Black African IAT. Participants were instructed to categorize words as quickly as possible while also being accurate. The IAT is composed of three practice blocks (omitted for analyses) and four critical blocks. We illustrate the measure by describing the critical blocks in the AV‐IAT for power‐universalism. In the first pair of critical blocks (20 trials and then 40 trials) following two practice blocks, participants categorized words related to power/universalism values and good/bad words on alternating trials. For example, participants may have categorized power values and good words with one key and universalism values and bad words with another key. (Half of the participants would have had the reverse key assignments.) In the second pair of critical blocks (20 trials and then 40 trials), participants categorized pairings opposite to the ones in the first pair of critical blocks. For each IAT, the two pairs of critical blocks were counterbalanced (between participants) to control for potential order effects, and the position of the “good/bad” categories was also randomized between participants.

The IATs were scored with the *D* algorithm recommended by Greenwald, Nosek, and Banaji (2003). A positive *D*‐score indicated faster responding on average when power values, achievement values, or French people were paired with good words and universalism values, benevolence values, or Black African people were paired with bad words compared with the reverse. Positive scores indicate a preference for power values compared to universalism values, a preference for achievement values compared to benevolence values, and a preference for French people compared to Black African people.

##### Split‐Half Correlations

To assess the internal consistency of the *D*‐IAT scores, we used the standard procedure: We calculated split‐half reliabilities over the differences scores of Block 6/3 and Block 7/4 (Schnabel, Asendorpf, & Greenwald, 2008). After applying the Spearman‐Brown correction, split‐half correlations for the power‐universalism AV‐IAT, *r*(82) = .76, *p* < .001, achievement‐benevolence AV‐ IAT, *r*(82) = .78, *p* < .001, and French–ethnic out‐group IAT, *r*(85) = .67, *p* < .001, were large and significant.

#### Results and Discussion

Table 1 presents the correlations between the three IATs. As in Study 1, spontaneous positivity to power (vs. universalism) was associated with more spontaneous negativity to the ethnic out‐group. In contrast, spontaneous positivity to achievement (vs. benevolence) was uncorrelated with spontaneous attitude to the ethnic out‐group.

A multiple regression analysis was conducted to test whether the power‐universalism AV‐IAT continued to predict spontaneous prejudice after controlling for achievement‐benevolence AV‐IAT scores. The total effect of both predictors was significant, *R* ^2^ = .08, *F*(2, 70) = 3.28, *p* = .04. As in Study 1, results indicated that the implicit measure of attitudes to power versus universalism continued to predict the French–out‐group IAT score (β = .32, *p* = .01, ${ŋ}_{p}^{2}$ = .08), but the implicit measure of attitudes to achievement versus benevolence (β = –.11, *ns*) did not. Thus, as expected, the power versus universalism AV‐IAT, but not the achievement versus benevolence AV‐IAT, again predicted spontaneous prejudice.

## Study 3

Numerous studies have shown that it is possible to change implicitly measured attitudes through classical conditioning (e.g., De Houwer, Baeyens, & Field, 2005; Olson & Fazio, 2001). In Study 3, we wanted to test whether the application of this approach to power and universalism values would influence spontaneous prejudice to Black African people. Extending the results of Studies 1 and 2, we expected that conditioning participants to form more positive associations with power values and more negative associations with universalism values would cause more spontaneous prejudice than conditioning participants to form opposite‐valenced associations with the values. This finding would provide support for the idea that there is a causal influence of these associations, which is an issue that could not be addressed in the correlational designs used for Studies 1 and 2.

It is worth noting that there is a lot of evidence that conditioning may also influence explicitly reported attitudes (Hofmann, De Houwer, Perugini, Baeyens, & Crombez, 2010). Nonetheless, the social undesirability of prejudice makes it difficult to assess the impact of conditioned associations on explicit measures of prejudice. Furthermore, our particular interest was in verifying the role of associations with power and universalism in spontaneous prejudice. Therefore, we did not explicitly measure prejudice in this study.

#### Method

##### Participants and Procedure

One hundred fifty‐nine participants took part in groups of 15 to 20 during lectures. The sample included 71 women and 88 men, with a mean age of 20.89 years (*SD* = 2.54). The experiment employed a three‐level (power‐positive/universalism‐negative vs. power‐negative/universalism‐positive vs. control) between‐subjects design. Participants first completed a paper‐and‐pencil flower‐insect IAT (Greenwald et al., 1998) as practice. Next, some participants were randomly assigned to a condition training them to associate either power values with positive adjectives and universalism values with negative adjectives (*n* = 53) or power values with negative adjectives and universalism values with positive adjectives (*n* = 52). A third group of control participants (*n* = 54) simply proceeded to the dependent measure, which was a paper‐and‐pencil French versus Black African IAT completed by all participants. The IATs were identical to those used in the previous studies, so we describe here only the experimental manipulation.

##### Experimental Manipulation

###### Power‐Positive/Universalism‐Negative Versus Power‐Negative/Universalism‐Positive Conditions

In the power‐positive/universalism‐negative condition, participants were given a page containing two columns. In the first column, 36 words related to one of the four categories (i.e., universalism, power, positive, and negative) were given in random order. Three items were presented for each of the four categories, with all items repeated three times. These items were the same as those used during the IATs in the previous studies. In the second column, which was left blank, participants wrote an item corresponding to a specific association. If the item in the left was related to “power” (e.g., *authority*), participants were asked write one of the three items related to “positive” (i.e., *wonderful*, *joyful*, *excellent*). If the item in the left column was related to “universalism” (e.g., *social justice*), participants were asked to choose and write one of the three items related to “negative” (i.e., *terrible*, *nasty*, *horrible*) in the right column. Also, if the item on the left was related to “positive” (e.g., *wonderful*), participants were asked to write one of the three items related to “power” (e.g., *wealth*), and if the item on the left was related to “negative” (e.g., *nasty*), participants were asked to write one of the three items related to “universalism” (e.g., *equality*). A similar procedure was employed in the power‐negative/universalism‐positive condition, except that participants paired power values with negative adjectives and universalism values with positive adjectives. The experimenter asked the participants to be as creative as possible by trying not using the same items repeatedly, but using equally all three items per category. This instruction helped to ensure that participants thought carefully about each response.

Participants completed this learning procedure across four blocks of trials in each of the two experimental conditions. In the first block, participants were given no time limit to complete the associations. Participants were given 2 minutes for the second block, 1 minute in the third block, and only 30 seconds in the last block. The increasing time constraints were intended to ensure that the associations became progressively more automatic.

#### Results and Discussion

The *D*‐scores shown in Table 1 reveal that participants' spontaneous evaluations of French people were significantly more positive than their spontaneous evaluations of Black African people. This pattern was replicated within each experimental condition. More relevant to our hypotheses, a three‐level between‐subjects ANOVA revealed that *D*‐scores significantly differed between conditions, *F*(2, 147) = 4.77, *p* < .01, ${ŋ}_{p}^{2}$ = .061. As expected, Newman‐Keuls post hoc analyses revealed that prejudice *D*‐scores were higher in the power‐positive/universalism‐negative condition (Table 1) than in the power‐negative/universalism‐positive condition (*p* < .01). This effect was symmetrical: Prejudice *D*‐scores in the control condition trended lower than *D*‐scores in the power‐positive/universalism‐negative condition (*p* = .08) and higher than *D*‐scores in the power‐negative/universalism‐positive condition (*p* = .17), but neither effect reached conventional levels of significance. Overall, the results indicate that associations with power and universalism values influence prejudice, converging with the correlational evidence yielded by the AV‐IATs in the first two studies.

## Study 4

Our fourth study tested whether the power‐universalism AV‐IAT predicts attitudes to a third important target group: women. Prior research has demonstrated the utility of implicit measures of gender attitude (Rudman & Kilianski, 2000; Skowronski & Lawrence, 2001), and we wished to test whether scores on these implicit measures can be predicted by the power‐universalism AV‐IAT. If greater spontaneous favorability to power over universalism values is associated with greater general favorability to dominant over nondominant groups, then people with higher scores on the power‐universalism AV‐IAT should be more likely to express greater spontaneous favorability to men over women.

Because spontaneous attitudes can be important predictors of spontaneous behavior (Fazio & Olson, 2003; Perugini et al., 2010; cf. Greenwald et al., 2009), we also wished to test whether the power‐universalism AV‐IAT could predict spontaneous gender‐related behavior. Here, we capitalized on our experience studying gender biases in sports referees. For example, we have found that referees in handball matches exhibit a particular style of bias against female players (e.g., Souchon et al., 2009; Souchon, Livingstone, & Maio, 2013). (Handball is a team sport that has been in the Olympic Games for over 40 years.) Referees of both genders award free throws more frequently to female victims of a foul (in games between women) than to male victims of a similar foul (in games between men), and the referees also punish female aggressors (in women's games) more severely than male aggressors (in men's games) who have committed the same type of foul. That is, aggression in women's handball games is censured more strongly than aggression in men's handball games–a pattern that has also been observed in soccer (Coulomb‐Cabagno, Rascle, & Souchon, 2005) and in basketball (Graf, Yabko, & Christensen, 2009). These biases are manifested without awareness and deliberation (e.g., under time pressure and cognitive busyness). We were therefore interested in testing whether this spontaneous bias is predictable by spontaneous evaluations of power and universalism values. Consequently, the main objective of Study 4 was to replicate the power‐universalism AV‐IAT's predictive validity by studying men's spontaneous gender attitudes and their quick decision making regarding ambiguous fouls during men's and women's handball matches, while under time pressure.

To further probe the AV‐IAT's discriminant validity, we assessed individual differences in hostile sexism (HS) and benevolent sexism (BS; Glick & Fiske, 1996). HS is an adversarial view of gender relations in which women are perceived as seeking to control men, whether through sexuality or feminist ideology, whereas BS is defined as a subjectively chivalrous ideology that offers protection and affection to women who embrace conventional roles. People who exhibit high HS and BS also attach significantly more importance to power values and less importance to universalism values (Feather, 2004). Thus, it was interesting to test whether HS and BS predict differences in spontaneous favorability to power and universalism values and, for the purpose of further establishing the unique predictive validity of the power‐universalism AV‐IAT, whether this measure predicts gender bias independently of any associations it has with HS or BS.

#### Method

##### Participants and Procedure

Participants examined video‐recorded events during handball games. Thus, we used handball players as participants because familiarity with this game and its rules were essential. We expected handball players to replicate the biases seen in referees (e.g., Frank & Gillovich, 1988; Souchon et al., 2013). Our sample comprised 95 experienced male handball players (*M*_age_ = 24.16, *SD* = 5.19; *M*_experience_ = 12.45 years, *SD* = 6.56) who competed on a local (*n* = 15), intermediate (n= 30), or national level (*n* = 50).

Participants completed (a) a refereeing decision task, (b) a set of paper‐and‐pencil implicit measures (practice IAT, power‐universalism AV‐IAT, achievement‐benevolence AV‐IAT, gender IAT), and (c) a set of explicit measures (attitudes to power, universalism, achievement, and benevolence values; sexism). The implicit and explicit measures of attitudes to values were the same as in Study 1. The measure of sexism was a validated French translation (Dardenne, Delacollette, Grégoire, & Lecocq, 2006) of the Ambivalent Sexism Inventory (ASI; Glick & Fiske, 1996), with 22 items (α = .83 for HS; α = .69 for BS).

The experimenter explained that the aim of the study was to better understand referees' decision making in handball games and that participants would also be asked to complete unrelated measures of attitudes for a different purpose. Finally, participants were probed for suspicion and debriefed.

##### Refereeing Task

Each participant watched two digital videos on a 1.42 × 1.88 m display. The principal content of each video comprised 10 handball situations with male players or 10 situations with female players. In each situation, the player in possession of the ball missed his or her shot at the goal after a transgression from a defensive player, and the transgressions were chosen to be very similar between male and female players. Using the first author's expertise on handball refereeing, we selected 72 pairs of very similar situations between male players and between female players from a large sample of videotaped games. For each of these pairs, 12 national referees answered the following two questions on a scale ranging from 1 (*absolutely not*) to 5 (*strongly similar*): (a) “In your opinion, are the two situations identical/strictly comparable as regards the decision‐making processes that are involved?” and (b) “Is the intensity of contact really identical/strictly comparable from your point of view?” Pairs of situations were selected only when there was near‐unanimous agreement between the 12 national referees, enabling us to be certain that male and female players in these situations should officiate the same way (see also Frank & Gillovich, 1988; Souchon et al., 2013). Moreover, the situations presented in the videos for men's and women's games were chosen to be very similar at each position in the sequence (i.e., first foul, second foul). For each participant, this presentation order and the order of the two videos were randomized.

For each of the 20 (10 × 2) situations, participants had to make two decisions. First, they decided whether or not to intervene, using a scale ranging from 1 (*absolutely certain that play should continue without intervention*) to 9 (*absolutely certain that the ball should be returned to the victim*). After making this decision for all situations, participants viewed the videos again and made disciplinary decisions regarding the offender, using a scale ranging from 1 (*absolutely certain that the defensive player does not have to be punished through a 2‐min exclusion*) to 9 (*absolutely certain that the defensive player has to be punished through a 2‐min exclusion*). For the decisions regarding players of both genders, the 10 intervention ratings and the 10 disciplinary ratings were averaged to form indices of the intervention decisions (α = .56) and the disciplinary decisions (α = .77). Higher scores indicated more certainty about intervention and greater punitiveness to the transgressor. Because participants were unaware that we were comparing responses to male and female players, the measurement of their decision making was indirect and, to their knowledge, not related to gender.

Before each video, participants were given four training situations, which were very similar across the videos containing male players and female players at each step in the presentation sequence. Following each situation (training and target), participants received a 2‐second countdown to make their refereeing decision. A sound effect after 2 seconds signaled the presentation of the next situation. This rapid pace created a realistic time pressure and prevented extensive thought about the decision, while allowing for the fact that participants were not experienced referees. In this way, the participants' decisions under time pressure were relatively spontaneous, but within enough time for them to make their decisions.

#### Results and Discussion

##### Implicit Measures

Mean AV‐IAT effects in our sample were calculated using the same procedure as in Studies 1 and 3. The *D*‐scores in Table 1 show that participants' spontaneous evaluations of power and universalism did not differ significantly. Similarly, participants' spontaneous evaluations of achievement and benevolence did not differ. As in Study 2, and congruent with Schwartz's (1992) model, participants who were more favorable to power on the power‐universalism AV‐IAT were more likely to show greater favorability to achievement on the achievement‐benevolence AV‐IAT (see Table 5). In addition, Table 1 shows that participants' spontaneous attitude to men was more positive than to women. Given that our sample was male, this finding is congruent with evidence that spontaneous gender attitudes tend to favor the in‐group (Rudman & Goodwin, 2004).

##### Explicit Measures

Table 4 indicates that participants expressed less positivity to power than to universalism, *t*(94) = −4.80, *p* < .001, and they endorsed achievement more than benevolence values, *t*(94) = 4.43, *p* < .001. Participants' hostile sexism scores were higher than their benevolent sexism scores, *t*(92) = 2.81, *p* < .01. Fitting Schwartz's (1992) model, participants who were more favorable to power were more favorable to achievement (see Table 5).

**Table 4:** Study 4: Descriptive Statistics for Explicit Measures

	ND	α	*M*	*SD*	95% CI
Power values	0	.92	.79	1.24	[.53, 1.04]
Universalism values	0	.93	1.56	1.11	[1.34, 1.79]
Power‐universalism values	0	–	−.77	1.56	[–1.08, −.45]
Achievement values	0	.87	2.52	.62	[2.39, 2.65]
Benevolence values	0	.91	2.10	.86	[1.92, 2.28]
Achievement‐benevolence values	0	–	.42	.92	[.23, .61]
Ambivalent sexism	2	.80	2.67	.66	[2.53, 2.81]
Hostile sexism	2	.83	2.82	.90	[2.64, 3.01]
Benevolent sexism	2	.69	2.51	.78	[2.35, 2.68]

*Note*. ND = number of deletions or missing data; 95% CI = 95% confidence interval.

**Table 5:** Study 4: Correlations Between Refereeing Bias and Implicit and Explicit Measures

	1	2	3	4	5	6	7	8	9	10	11	12	13
1. PU IAT	–	.24+	.31*	.27*	.09	−.46**	.39**	.11	−.14	.21	.02	.06	.05
2. AB IAT		–	−.02	−.06	.06	−.12	.14	.10	−.21+	.23+	.15	.19	.21+
3. G IAT			–	−.05	.02	−.15	.12	.02	−.21*	.21*	.13	.14	.17+
4. Gen bias				–	−.09	.08	−.12	.01	−.04	.05	.03	−.05	−.01
5. Pow					–	.11	.77**	.33**	.10	.12	.16	.15	.20+
6. Uni						–	−.61**	.04	.33**	−.28*	.02	.15	.10
7. Pow‐Uni							–	.23*	−.15	.30**	.11	.01	.08
8. Achi								–	.26*	.42**	.08	.24*	.20+
9. Ben									–	−.75**	−.10	.26*	.08
10. Achi‐Ben										–	.15	−.08	.06
11. HS											–	.22*	.82**
12. BS												–	.74**
13. AS													–

*Note*. PU IAT = power‐universalism AV‐IAT; AB IAT = achievement‐benevolence AV‐IAT; G IAT = gender attitude IAT; Gen bias = general refereeing bias; Pow, Uni, Achi, and Ben = explicit measures of attitude toward power, universalism, achievement, and benevolence; HS = hostile sexism; BS = benevolent sexism. *N*s vary between 56 and 95 due to missing data on the power‐universalism AV‐IAT, the achievement‐benevolence AV‐IAT, and the gender IAT.

+*p* < .10. *p < .05. **p < .01.

##### Implicit‐Explicit Correlations

###### Correlations Between Related Constructs Measured Implicitly or Explicitly

Table 5 indicates that participants who scored higher on the power‐universalism AV‐IAT expressed both lesser favorability to universalism and greater favorability to power over universalism on the explicit measure. Similarly, participants who scored higher on the achievement‐benevolence AV‐IAT expressed both lesser favorability to benevolence and greater favorability to achievement over benevolence on the explicit measure. Participants who scored higher on the gender IAT did not express significantly higher hostile and benevolent sexism, consistent with other evidence of discrepancies between IAT measures of in‐group bias and self‐reports of such bias (Greenwald & Nosek, 2009).

###### Predicting Spontaneous Gender Bias

As expected, spontaneous favorability to power versus universalism predicted spontaneous positivity to men over women (see Table 1). In contrast, as described in Table 5, explicit favorability to power or universalism did not predict spontaneous attitudes to men over women. This spontaneous gender bias was not significantly predicted by scores on the achievement‐benevolence AV‐IAT, but it was unexpectedly linked to explicit favorability to benevolence.

###### Predicting Hostile and Benevolent Sexism

As shown in Table 5, participants who scored higher on the power‐universalism AV‐IAT did not express higher hostile or benevolent sexism. Similarly, participants who scored higher on the achievement‐benevolence AV‐IAT did not manifest significantly more hostile or benevolent sexism.

##### Prediction of Gender Bias in Refereeing Task

As in past research (e.g., Souchon et al., 2009, 2013), our male participants were significantly more likely to intervene and discipline female players (*M* = 44.88, *SD* = 7.08) than male players (*M* = 42.9, *SD* = 8.47), across all cells of the 2 (video order) × 3 (competition level) × 2 (type of decision) mixed‐model design, *F*(1, 89) = 5.42, *p* = .02, ${ŋ}_{p}^{2}$ = .06 (without significant reversals in any cell). The degree of gender bias was calculated for each participant by subtracting the intervention scores for the male players from the intervention scores for the female players and by subtracting the disciplinary scores for the male players from the disciplinary scores for the female players. Gender bias was calculated as the mean across both types of bias, *r*(95) = .25, *p* = .02. Positive scores on these indices revealed that participants were more severe against fouls made by female players than by male players. As shown in Table 5, participants who were spontaneously more favorable to power versus universalism, but not participants who were spontaneously more favorable to achievement versus benevolence, exhibited more gender bias in the officiating decisions. The explicit measures of attitudes to values and sexism did not significantly predict the gender bias.

##### Regression Analyses Predicting Gender Attitude and Decision Bias

Similar to Study 1, a regression analysis, *R* ^2^ = .20, *F*(4, 44) = 2.73, *p* = .04, indicated that the power versus universalism AV‐IAT continued to predict the gender IAT score (β = .42, *p* < .01, ${ŋ}_{p}^{2}$ = .14), whereas the achievement versus benevolence AV‐IAT (β = –.24, *ns*) and the explicit measure of attitudes to power versus universalism (β = –.04, *ns*) and achievement versus benevolence (β = .17, *ns*) did not. Also, in a second regression analysis entering the gender bias against women in the refereeing task as the criterion, the power‐universalism AV‐IAT marginally predicted the general bias (β = .34, *p* = .06, *R* ^2^ = .11), but the other implicit (achievement‐benevolence AV‐IAT, gender IAT) and explicit measures (attitude to power vs. universalism and achievement vs. benevolence) did not. However, the overall model in this analysis was not reliable, *F*(5, 43) = 1.01, *p* = .42, because of the inclusion of many weak predictors. We therefore conducted two regression analyses that included the power‐universalism AV‐IAT alongside the other explicit *or* implicit measures. The power versus universalism AV‐IAT continued to predict the gender bias (β = .38, *p* < .01, ${ŋ}_{p}^{2}$ = .12) after controlling for the explicit measures of attitude to power versus universalism (β = –.22, *ns*) and achievement versus benevolence (β = –.11, *ns*), overall *R* ^2^ = .14, *F*(3, 54) = 2.99, *p* = .03. The power‐universalism AV‐IAT continued to predict the gender bias (β = .35, *p* = .01, ${ŋ}_{p}^{2}$ = .12) after controlling for the achievement versus benevolence AV‐IAT (β = –.11, *ns*), *R* ^2^ = .12, *F*(2, 48) = 3.24, *p* = .04.

#### Summary

As expected, the power versus universalism AV‐IAT uniquely predicted spontaneous attitudes to gender *and* gender bias against women in the refereeing task. Thus, the implicit measure of attitudes to these values again explained variance in spontaneous judgments and action that could not be accounted for by the other measures.

## Study 5

Our final study tested whether the power‐universalism AV‐IAT correlates with a new power‐universalism IAT that focuses on importance judgments and whether both predict implicitly measured prejudice. As outlined in our introduction, values are theoretically defined according to an importance dimension (e.g., Schwartz, 1992), but these importance judgments are strongly linked to affective responses to values (see Maio, 2010, for a review). Because of the lack of an empirical precedent for assessing judgments of importance with an implicit measure, we were less confident that implicit measures of value importance could tap these affective responses to values effectively. Nonetheless, the AV‐IAT should tap these affective associations, which should also predict the implicit measurement of importance attached to values *if* both types of implicit measure converge on these associations.

Also, to provide a stricter test of the unique validity of the AV‐IATs, the previous studies used an explicit measure of attitude to power and universalism, instead of an explicit measure of value importance. This approach enabled the implicit and explicit measures to assess the same type of judgment (i.e., attitudinal evaluations) instead of different types of judgment (i.e., evaluations vs. importance). Study 5 also included a standard explicit measure of value importance. We expected that both the AV‐IAT for power versus universalism values and the new implicit measure of the importance of these values would predict implicit prejudice, over and above the explicit importance of power versus universalism values.

#### Method

##### Participants and Procedure

One hundred thirty‐six participants (48 women; *M*_age_ = 19.95, *SD* = 2.50) completed the study in groups of 10 to 25 within university. All participants were of French nationality, but 13 participants had at least one parent originating from a Black African country and were therefore excluded from analyses. Participants were told that they were taking part in a study of personality. They completed a set of paper‐and‐pencil implicit measures (practice IAT, power‐universalism AV‐IAT, importance vs. unimportance power‐universalism IAT, French vs. Black African IAT) and a set of explicit measures (attitudes to power, universalism, French people, and Black African people; importance attached to power and universalism). The implicit and explicit measures of attitudes to values and to Black African people were the same as in previous studies, so only the implicit and explicit measures of value importance are described below. Finally, participants were debriefed.

##### Implicit and Explicit Measures of Value Importance

The implicit measure of the importance attached to power versus universalism values used the same structure as the evaluative IATs, except that the stimuli did not include positive and negative adjectives, but items that were related to importance (i.e., *important*, *essential*, *fundamental*) and unimportance (e.g., *unimportant*, *secondary*, *insignificant*). For the explicit measures of importance, participants completed a shortened version of the Schwartz Value Survey (Schwartz, 1992). This version contained the same power (i.e., authority, social power, and wealth) and universalism values (equality, broad‐mindedness, and social justice) used in the implicit measure, with two openness values (i.e., freedom and stimulating life) and two conservation values (i.e., obedience, faith). In this survey, each of the 10 values was printed beside a definition of the value (e.g., equality, equal opportunity for all). Participants were asked to rate each value in terms of its importance as a guiding principle in their life, using the 9‐point scale recommended by Schwartz (1992): −1 (*opposed to my values*), 0 (*not important*), 3 (*moderately important*), 6 (*very important*), and 7 (*extremely important*).

#### Results and Discussion

##### Implicit Measures

Table 1 presents descriptive data for the IATs. As in the previous studies, the *D*‐scores and *t*s show that participants in this study exhibited significantly less spontaneous favorability to power than to universalism and attached less spontaneous importance to power than to universalism values. The correlation between both value‐focused implicit measures was moderate in size and significant. As in Studies 1–3, the implicit measure of prejudice found more negativity to Black African people than to French people.

##### Explicit Measures

Table 6 presents the descriptive statistics for the explicit measures. Participants explicitly evaluated power less favorably than universalism, *t*(122) = −12.04, *p* < .001, and attached less importance to power values than universalism values, *t*(122) = −5.21, *p* < .001. Participants were as favorable to people of French origin as to Black African people, *t*(122) = –.80, *p* > .05. As expected, the correlations between both value‐focused explicit measures were moderated in size and significant (see Table 7).

**Table 6:** Study 5: Descriptive Statistics for Explicit Measures

Attitude Measures	ND	α	*M*	*SD*	95% CI
					
Power values	0	.91	−.004	1.09	[−.19, .19]
Universalism values	0	.93	1.69	1.05	[1.51, 1.88]
Power‐universalism values	0	–	−1.7	1.56	[–1.97, −1.42]
French	0	.96	1.17	1.21	[.95, 1.38]
Black African	0	.97	1.21	1.24	[.99, 1.43]
French–Black African	0	–	−.04	.60	[−.15, .06]
Importance measures					
Power values	0	.73	−.24	2.48	[−.69, .19]
Universalism values	0	.67	1.26	.99	[1.09, 1.44]
Power‐universalism values	0	–	−1.51	3.21	[–2.09, −.93]

*Note*. ND = number of deletions or missing data; 95% CI = 95% confidence interval.

**Table 7:** Study 5: Correlations Between Implicit and Explicit Measures

	1	2	3	4	5	6	7	8	9	10	11	12
1. PU AV‐IAT	–	.46**	.24*	−.03	−.18+	.09	−.08	−.07	−.04	−.11	−.09	−.03
2. PU Imp IAT		–	.27**	−.03	−.18+	.10	−.05	−.13	−.01	.01	−.01	.02
3. Fr‐Black IAT			–	.12	−.14	.17+	.03	−.17+	.07	.12	.06	.13
4. Ex Att Pow				–	−.06	.73**	.36**	−.49**	.43**	−.05	−.13	.15+
5. Ex Att Uni					–	−.71**	−.12	.38**	−.21*	.16+	.17+	−.02
6. Ex Att Pow‐Uni						–	.33**	−.60**	.44**	−.14	−.20*	.12
7. Ex Imp Power							–	−.66**	.97**	.05	.02	.06
8. Ex Imp Uni								–	−.80**	.07	.10	−.06
9. Ex Imp Pow‐Uni									–	.01	−.01	.06
10. Ex Att French										–	.87**	.19*
11. Ex Att Black‐A											–	−.29**
12. Ex Att Fr‐B												–

*Note*. PU AV‐IAT = power‐universalism AV‐IAT; PU Imp IAT = importance power‐universalism IAT; Fr‐Black IAT = implicit attitude toward French versus Black African people; Ex Att Pow, Ex Att Uni, Ex Att Pow‐Uni, Ex Att French, Ex Att Black‐A, and Ex Att Fr‐B = explicit measures of attitude toward power, universalism, French people, Black African people, power versus universalism, and French people versus Black African people; Ex Imp Power, Ex Imp Uni, and Ex Imp Pow‐Uni = explicit importance attached to power, universalism, and power versus universalism. *N*s vary between 86 and 123 due to missing data on the IATs.

+*p* < .10. *p < .05. **p < .01.

##### Prediction of Prejudice

Table 7 presents the correlations among all our measures. Both higher spontaneous positivity to power (vs. universalism) and higher spontaneous importance to power (vs. universalism) were associated with a spontaneous negative attitude to the ethnic out‐group. Looking at the explicit measures, participants who evaluated power more favorably in relation to universalism were more negative to Black African people. Turning to the implicit‐explicit correlations, results again suggested some independence between the implicit and explicit measures of values: Both the implicit measurement of attitudes to power versus universalism and the implicit importance attached to power versus universalism were at best weakly associated with the corresponding explicit attitudes and importance judgments for the values.

A regression analysis revealed that the power‐universalism AV‐IAT predicted spontaneous attitude to Black African people (β = .24, *p* < .01), whereas explicit power‐universalism measures did not (βs ≤ .13, *ps* ≥ .29), overall *R* ^2^ = .10, *F*(3, 89) = 3.13, *p* = .02. In addition, the power‐universalism importance IAT continued to predict spontaneous attitude to Black African people (β = .26, *p* = .009, ${ŋ}_{p}^{2}$ =.07), whereas the explicit power‐universalism measures did not (βs ≤ .09, *p*s ≥ .38), overall *R* ^2^ = .10, *F*(3, 95) = 3.29, *p* = .02. Finally, the power‐universalism AV‐IAT did not predict spontaneous attitude to Black African people (β = .20, *p* = .10) after controlling for the explicit power‐universalism measures and the implicit power‐universalism IAT (β = .16, *p* = .20). Thus, the predictive ability of the values AV‐IAT may be partly attributable to shared variance with the values‐importance IAT.

## General Discussion

Although research studies have frequently examined factors that attenuate or augment value‐attitude‐behavior relations, studies have not considered the role of spontaneous associations with values. Across five studies and six samples, we predicted and found that spontaneous favorability to two important values, power and universalism, predicted greater spontaneous positivity to an ethnic in‐group over ethnic out‐groups (Studies 1, 2, 3, and 5), and to men over women (Study 4). Moreover, spontaneous favorability to power over universalism values uniquely predicted gender bias in a refereeing decision task (Study 4). Finally, spontaneous attitude to power versus universalism correlated strongly with the spontaneous importance ascribed to the values, and both measures predicted an implicit measure of prejudice (Study 5).

Notwithstanding these consistent results, we should note that each separate study was slightly underpowered to detect small‐to‐medium effect sizes. Consequently, to probe the robustness of the associations between the power‐universalism AV‐IAT and the implicit measures of prejudice across the six samples, we conducted a meta‐analysis (*N* = 476). This analysis was conducted in the program *R*, using formulas developed by Borenstein, Hedges, Higgins, and Rothstein (2009) to test a random effects model, wherein the true effect size may vary from study to study (as the samples and target groups differed). (In all of our analyses, the results did not differ if a fixed effects model was assumed instead of a random effects model.) The results indicated a mean of *r* = .26, 95% CI [.17, .34], *Z* = 5.56, *p* < .0001, and heterogeneity of the true effect sizes was not observed, *Q*[5] = 0.70, *p* = .98. In contrast, the summary effect for the achievement‐benevolence AV‐IAT was nonsignificant across the three studies that included it, *r* = .08, 95% CI [–.06, .22], *p* = .25. Furthermore, although the association between explicit favorability to power (vs. universalism) and the implicit measure of prejudice (Studies 1, 4, and 5; *N* = 365) was reliable, 95% CI [.02, .23], *p* = .02, this was due to the large sample, as the effect was weak, *r* = .12, and less than half of the magnitude of the effect for the AV‐IAT. In fact, using Williams's (1959) *t*‐test for depending correlations, which has been shown to do better in terms of Type I and II error rates compared to similar tests (May & Hittner, 1997), the power‐universalism AV‐IAT was more strongly correlated with spontaneous prejudice compared to both the achievement‐benevolence AV‐IAT, *t*(269) = 3.02, *p* = .001, and explicit favorability to power (vs. universalism), *t*(289) = 2.14, *p*= .02.^3^

To our knowledge, this research is the first demonstration that spontaneous value associations help to predict spontaneous attitudes and behavior. Past research on values has used explicit measures like the Schwartz Values Survey (Schwartz, 1992), but the present data show that an implicit measure of attitudes to values or an implicit measure of importance (Study 5) may also be useful when used alternatively or in combination. Measuring spontaneous associations to values constitutes an easy way to capture the affect that individuals attach to values without asking them to introspect. It would be easy to expand this approach to assess other values described in Schwartz's (1992) model, and this might help to predict diverse judgments and behaviors. For example, spontaneous preferences between stimulation versus security values may be uniquely related to spontaneous associations with alcohol, drugs, and calorie‐rich food. This is an interesting issue because Bar‐Anan and Nosek (2014) found that associations between different implicit measures regarding the same attitude object depend on the attitude domain (e.g., race, politics, and self‐esteem). It is important to compare relations between implicit measures of values and attitudes and behavior in diverse domains.

Although the AV‐IAT we developed provided consistent support for the utility of measuring spontaneous associations with values, it is important to recognize that the IAT is not the only useful method for implicit measurement. Other implicit measures take different but useful approaches. For example, the personalized IAT looks at spontaneous associations personally endorsed by participants, rather than being open to extrapersonal associations, such as the influence of culture and media (Olson & Fazio, 2004; see also Fiedler et al., 2006; Teige‐Mocigemba et al., 2010). Our reasoning was that the extrapersonal associations are important, even if not personally endorsed, because they reflect the environment in which the values operate (see also Nosek & Hansen, 2008). At the same time, however, the personalized associations may play their own unique roles. This and other implicit techniques merit future research. For instance, another approach is to use single‐category IATs (SC‐IAT; Karpinski & Steinman, 2006). SC‐IATs can focus on one value set at a time (e.g., power), which may help to detect whether one end of a value dimension is particularly important in the obtained effects. As indicated in our introduction, the oppositional motives postulated by Schwartz's (1992) model provided a theoretical basis for using the standard IAT, but the personalized IAT and the SC‐IAT may provide useful next steps.

It would also be interesting to compare these techniques with other procedures that have been used in the personality literature to examine individual differences in social motivation. It would be particularly interesting to consider measures of the *need* for power (see Winter et al., 1998; Schultheiss, 2008), which has been examined using explicit and implicit techniques (McClelland et al., 1989). Values and needs may influence each other (see Schwartz & Bardi, 1997), making it useful to examine their interaction using implicit and explicit measures of both constructs.

In sum, the thrust of our findings is that it is useful to expand the assessment of values beyond the self‐report measures already employed. This expanded assessment will help to complement the existing measures in a way that provides a broader base for understanding values and their impacts. Our evidence repeatedly shows that this approach can have predictive utility–many associations between power and universalism values and prejudice would have gone undetected using the explicit measures alone. The present findings help us better understand the power‐oriented behavior of the Napoleons and Machiavellis in our world, but an expansion of this approach may help us understand social behavior more generally.

### Declaration of Conflicting Interests

The authors declared no potential conflicts of interest with respect to the research, authorship, and/or publication of this article.

### Funding

The authors disclosed receipt of the following financial support for the research, authorship, and/or publication of this article: Preparation of this manuscript was supported by Grant ES/J500197/1 from the Economic and Social Research Council (ESRC; http://www.esrc.ac.uk) to the third author.

## Notes

1 These include Angola, Benin, Burkina Faso, Cameroon, Comoros, Ivory Coast, Equatorial Guinea, Ethiopia, Gabon, Ghana, Guinea, Madagascar, Mali, Niger, Senegal, South Sudan, Tanzania, Chad, Togo, and Zimbabwe.

2 It has been suggested that participants be excluded from analyses if they fail to classify at least eight items per page and made more than 20% errors per page in paper‐and‐pencil IATs (e.g., Lane et al., 2005). These criteria were consistently applied across our studies, and the number of deletions, ranging from 2.73% to 37.8%, is concordant with the number of deletions in Teachman and Brownell's (2001) use of the paper‐and‐pencil IAT (17.2% in mean across their two IATs) and the number of deletions in Lemm, Lane, Sattler, Khan, and Nosek's (2008) use of the paper‐and‐pencil IAT (e.g., 33% in study 2a with 8 items by page and 20% errors).

3 A regression analysis across Studies 1, 4, and 5, *r* ^2^ = .08, *F*(3, 278) = 7.75, *p* < .001, revealed that the implicit power‐universalism AV‐IAT continued to predict implicit prejudice (*β* = .24, *p* < .001) after controlling for the impact of the separate explicit measures of attitude toward power (β = .01, *p* = .77) and universalism (β = –.08, *p* = .16) instead of their difference score. This result shows that the superiority of the implicit measure did not arise because the use of the difference score in the explicit power‐universalism measure masked asymmetric effects of these values.

## Appendix A

**Table 1A:** Summary of Implicit Categories and Stimuli Across the Studies

	*Stimuli to be classified*				
*Target*	*Pen and paper IATs*			*+ Items computer IATs*	
Flowers	Daffodil	Daisy	Tulip		
Insects	Bugs	Mosquito	Roach		
French	Florent	Ludovic	Jérôme	Sophie	Julie
Arab	Samir	Rachid	Karim		
Black African S1	Mamadou	Ousmane	Cheikh	Diama	Coumba
Black African S5	Ibra	Samba	Adama		
Men	Pierre	Paul	Jacques		
Women	Marie	Sophie	Isabelle		
Power	Authority	Social Power	Wealth	To command	To manage
Universalism	Equality	Broad‐mindedness	Social Justice	World at peace	Tolerance
Achievement	Ambitious	Successful	Influential	Hard working	Social recognition
Benevolence	Forgiving	Helpful	Honest	Sincerity	Loyalty

Attribute	Pen and paper IATs	+ Items computer IATs
Bad	Terrible, Nasty, & Horrible	Tragic, Agony, Humiliating, Unpleasant, Clumsy
Good	Wonderful, Joyful, & Excellent	Fantastic, Pleasure, Glorious, Superb, Extraordinary
Importance	Important, Essential, & Fundamental	
Unimportance	Unimportant, Secondary, & Insignificant	

*Note. “*S1” is for Study 1, and “S5” is for Study 5.
